# Connecting Circadian Genes to Neurodegenerative Pathways in Fruit Flies

**DOI:** 10.1371/journal.pgen.1005266

**Published:** 2015-06-11

**Authors:** Patrick Emery

**Affiliations:** Department of Neurobiology, University of Massachusetts Medical School, Worcester, Massachusetts, United States of America; Washington University Medical School, UNITED STATES

Life expectancy has dramatically increased in recent human history. As a result, neurodegenerative diseases have become a primary health issue. Circadian clocks are found in all mammalian tissues, including neurons and glia, and are, thus, likely to have an impact on the onset and progression of neurodegenerative diseases. However, the link between circadian clocks and neurodegeneration is poorly understood. Means et al. now report an intriguing connection between circadian genes and pathways implicated in neurodegenerative processes [1].

Circadian rhythms are fundamental adaptive mechanisms that enable organisms to optimize most of their bodily functions with the day/night cycle. A critical circadian output in animals is the sleep/wake cycle. Circadian and sleep disruptions are frequently associated with neurodegenerative diseases [2]. Furthermore, sleep abnormalities can precede the onset of other neurological symptoms. For example, patients carrying a pathogenic allele of Ataxin-2 causing Spinocerebellar Ataxia Type 2 (SCA-2) experience sleep disruptions prior to suffering from ataxic symptoms [3,4]. Interestingly, in fruit flies, the ATXN2 homolog dATX2 plays a critical role in the expression of the circadian gene *period* (*per*) in circadian pacemaker neurons. Indeed, dATX2 collaborates with the translation factor TWENTYFOUR (TYF) to promote *per* mRNA translation [5,6]. Thus, the homolog of a gene involved in neurodegeneration contributes to the control of circadian rhythms in fruit flies. But what about the opposite connection: does the clock, or at least some circadian genes, contribute to the onset and progression of neurodegenerative diseases? Means et al. also turned to *Drosophila* to try to answer this important question [1].

The Price lab has had a long interest in a critical circadian kinase called DOUBLETIME (DBT), which is the fly homolog of Casein Kinase 1 δ/ε. DBT regulates PER phosphorylation, as well as the activity of the circadian transactivator CLOCK (CLK) [7]. DBT is, thus, critical to determining the period of circadian rhythms. Looking for DBT regulators, Means et al. identified SPAGHETTI (SPAG). SPAG downregulation leads to a long period phenotype or to arrhythmic behavior. SPAG has recently been shown to be part of a multimeric co-chaperone that works with HSP70 and HSP90 to regulate the assembly of large protein complexes [8]. HSP proteins play an important role in the progression of neurodegeneration [9]. Moreover, SPAG was found to affect aggregation of Huntingtin (HTT), the protein that causes Huntington disease when its polyQ domain is expanded [10].

**Fig 1:**
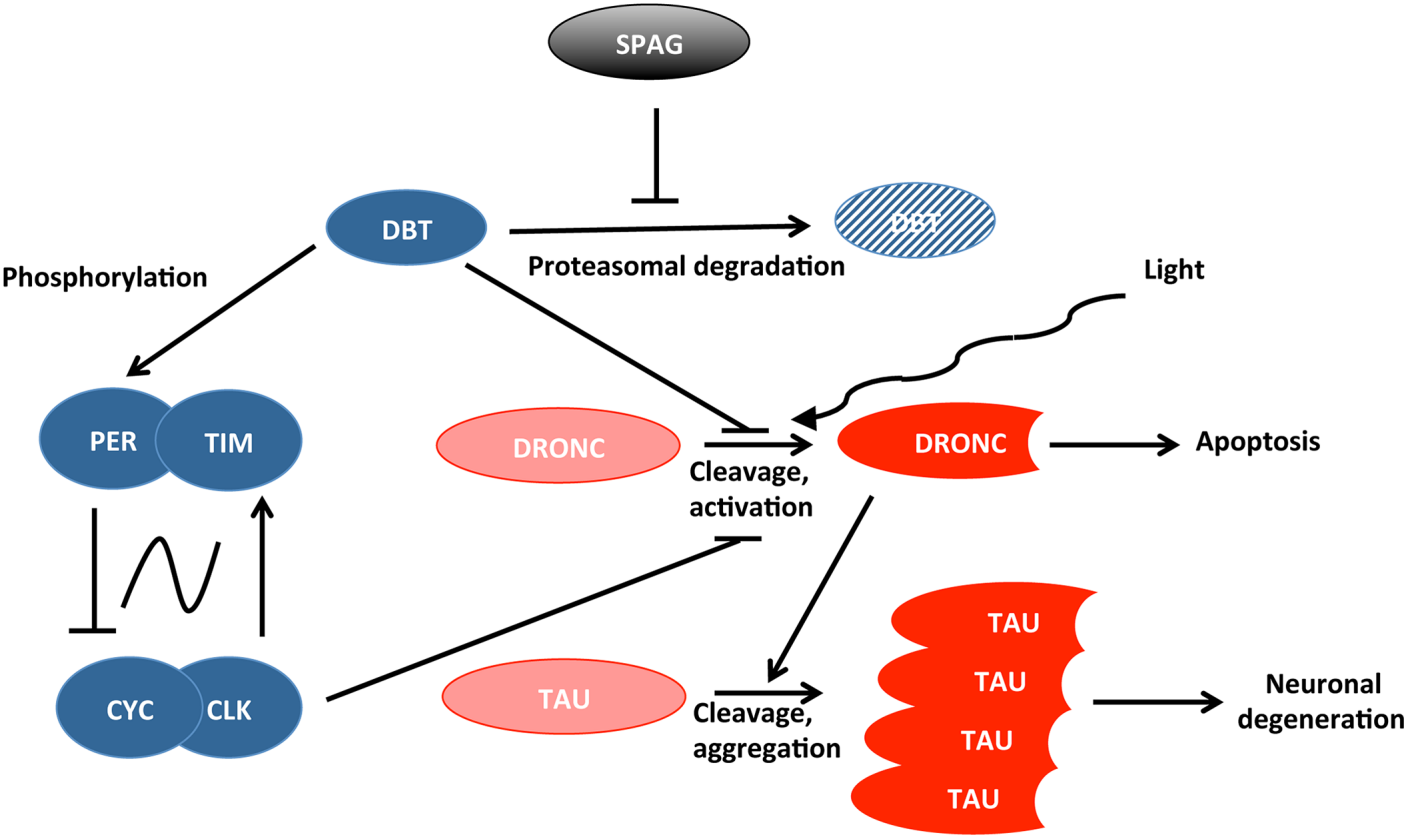
Connection between circadian proteins (in blue) and proteins causing cell death or neuronal degeneration (in red). SPAG and light modulate this protein network.

Means et al. uncovered a novel pathway connecting SPAG to neurodegenerative mechanisms in flies, which is modulated by circadian genes and light (Fig 1). Their results indicate that SPAG protects DBT from proteasomal degradation at specific time points: during the day about seven hours after the lights are turned on, and seven hours into the night. DBT disappearance during the day is very closely associated with activation of the caspase DRONC [11,12], but not at night. However, light exposure during the night can also cause DRONC activation, demonstrating that both light and loss of DBT are required. DRONC promotes cell death, and Means et al. show that it can also trigger TAU cleavage, which is observed in Alzheimer disease [13]. Loss of DBT also worsened the neurodegenerative phenotype observed when human TAU is overexpressed in the fly eye. Thus, SPAG and DBT protect flies against activation of caspases that can ultimately lead to neuronal degeneration and cell death.

Interestingly, the SPAG pathway appears to be entirely cell-autonomous since it can be recapitulated beginning from SPAG inactivation to TAU cleavage in *Drosophila* S2 cell culture. However, in the brain, a more complicated mechanism is at play. Indeed, DRONC activation is very broad in the brain, even when SPAG/DBT are inactivated in only 16 circadian neurons called ventral lateral neurons (LNvs), which express the neuropeptide Pigment Dispersing Factor (PDF). Moreover, the receptor for PDF (PDFR) is required for broad DRONC activation, which, in the brain at least, is largely non-autonomous. How a similar set of proteins can be involved in what appears to be rather different mechanisms of DRONC activation will need to be determined. Also surprising is the fact that downregulating DRONC only in tissues expressing circadian rhythms in the brain (clock neurons and glia) appears to block DRONC activation in non-circadian neurons. It seems that DRONC expression in circadian neurons is required for the spread of its activation. These are very intriguing observations that certainly warrant further study since this could shed light on how neurodegeneration spreads to large regions of the brain.

To come back to the initial question, what is the actual role of the circadian clock in this process? *In vivo*, DRONC activation implicates DBT, a key circadian protein, and PDF positive circadian neurons. It would, therefore, seem likely that the circadian molecular pacemaker is implicated. Unexpectedly however, although a strong dominant negative *clk* mutant caused DRONC to be activated, a null *per* mutation—which makes flies completely arrhythmic—did not. Moreover, DRONC activation happened in *clk* mutant flies at the same time as in wild-type flies. Finally, short and long period *per* mutants had no effect on the phase of DRONC activation. Thus, although at least two circadian proteins (CLK, DBT) control DRONC activation, it does not appear that the circadian molecular clock itself impacts this process. This conclusion could seem particularly unexpected since well-characterized circadian neurons and their neuropeptidic output PDF are critical for DRONC activation in the brain. However, there are two types of PDF positive circadian neurons. The small LNvs are the actual pacemaker for circadian behavior, which means that they determine the pace and phase of circadian locomotor rhythms [14]. Then there are the large LNvs, which have been shown to mediate various behavioral light responses, including acute light-induced arousal [15–17]. These neurons send numerous projections into the optic lobe and, thus, probably receive light input from the eyes. Moreover, they are themselves directly acutely light sensitive through the photoreceptor CRYPTOCHROME [18]. It seems therefore plausible that prolonged light exposure would cause the large LNvs to secrete PDF and, thus, trigger the broad activation of DRONC when the protective SPAG/DBT pathway is disrupted. Importantly, Means et al. show that this protective pathway is defective in aging wild-type flies, with DRONC becoming activated even under dark conditions. Neuronal aging in flies could be linked to SPAG/DBT pathway disruption. Whether similar mechanisms are at play in aging or diseased mammalian neurons now needs to be determined.
